# Branched-chain amino acids linked to depression in young adults

**DOI:** 10.3389/fnins.2022.935858

**Published:** 2022-09-30

**Authors:** Alyce M. Whipp, Marja Heinonen-Guzejev, Kirsi H. Pietiläinen, Irene van Kamp, Jaakko Kaprio

**Affiliations:** ^1^Institute for Molecular Medicine Finland (FIMM), University of Helsinki, Helsinki, Finland; ^2^Clinicum, Department of Public Health, University of Helsinki, Helsinki, Finland; ^3^Obesity Research Unit, Research Program for Clinical and Molecular Metabolism, Faculty of Medicine, University of Helsinki, Helsinki, Finland; ^4^Abdominal Center, Obesity Center, Endocrinology, University of Helsinki and Helsinki University Central Hospital, Helsinki, Finland; ^5^National Institute for Public Health and the Environment (RIVM), Bilthoven, Netherlands

**Keywords:** biomarkers, depression, valine, leucine, branched-chain amino acid, young adult mental health

## Abstract

Depression is a heterogeneous mental health problem affecting millions worldwide, but a majority of individuals with depression do not experience relief from initial treatments. Therefore, we need to improve our understanding of the biology of depression. Metabolomic approaches, especially untargeted ones, can suggest new hypotheses for further exploring biological mechanisms. Using the FinnTwin12 cohort, a longitudinal Finnish population-based twin cohort, with data collected in adolescence and young adulthood including 725 blood plasma samples, we investigated associations between depression and 11 low–molecular weight metabolites (amino acids and ketone bodies). In linear regression models with the metabolite (measured at age 22) as the dependent variable and depression ratings (measured at age 12, 14, 17, or 22 from multiple raters) as independent variables [adjusted first for age, sex, body mass index (BMI), and additional covariates (later)], we initially identified a significant negative association of valine with depression. Upon further analyses, valine remained significantly negatively associated with depression cross-sectionally and over time [meta-analysis beta = −13.86, 95% CI (−18.48 to −9.25)]. Analyses of the other branched-chain amino acids showed a significant negative association of leucine with depression [meta-analysis beta = −9.24, 95% CI (−14.53 to −3.95)], while no association was observed between isoleucine and depression [meta-analysis beta = −0.95, 95% CI (−6.00 to 4.11)]. These exploratory epidemiologic findings support further investigations into the role of branched-chain amino acids in depression.

## Introduction

Depression is a broad and heterogenous mental health problem, which affects 256 million adults and 21 million adolescents worldwide (GHDx *Institute of Health Metrics and Evaluation*, 2019; last accessed 9Feb2022). In 2019, for the global population of 10–24 and 25–49 year-olds, depression was the fourth and sixth leading causes of disability-adjusted life years (DALY), respectively (Diseases and Injuries, 2020). Depression can be severe and debilitating, with an increased risk of suicide, while in some cases, symptoms are mild and self-limiting. Unfortunately, about two-thirds of individuals with depression do not experience remission after initial treatment, and the prognosis is worse for those not responding to the initial treatment (Murrough and Charney, 2012). Overall episodes of depression can be recurrent, while some persons experience only one or a few episodes in their lifetime (Steinhausen et al., 2006; Pelkonen et al., 2008). Thus, we need to improve our understanding of depression and its biology to identify new markers of diagnosis and prognosis and new targets for drug treatment development.

The biology of depression has been a focus of investigation for decades, and while much has been discovered, we still require new approaches to elucidate mechanisms. Reviews of depression indicate at least five major biological systems are involved in depression, namely, inflammation, neuroendocrine, neurotransmitter, growth factor, and metabolic (Strawbridge et al., 2017; Zwolinska et al., 2021); biomarkers in these areas have been investigated using many approaches, including genetics (Flint and Kendler, 2014), epigenetics (Dalton et al., 2014), metabolomics (Bot et al., 2020), and the gut microbiome (Qin et al., 2022). Metabolomic investigations have been used in studies of somatic health conditions for many years and have more recently been used in psychiatry (Martins-de-Souza, 2014; Guest et al., 2016; Bot et al., 2020; Pu et al., 2021). Metabolomic approaches—-in particular untargeted approaches, which use a broad panel of metabolite information to investigate associations—-have been harnessed to generate new ideas and hypotheses in mental health (Bot et al., 2020; Whipp et al., 2021b), and continue to be useful in studies of depression.

Most depression biomarker investigations have used clinical datasets and/or adult samples (Baranyi et al., 2016; Zheng et al., 2017; Bot et al., 2020), while population-based cohorts and younger age samples are less common (Zwolinska et al., 2021). Since there appears to be a distinction between juvenile-onset and adult-onset depression (Jaffee et al., 2002), there is a strong need to investigate depression in both young and adult populations. In young adult samples, biomarkers could be either pre-diagnostic or post-onset, so the more ages involved in the search for depression biomarkers, the better we can distinguish biological differences in such a heterogenous mental health problem.

Depression is often found as a comorbidity with other psychopathologies (e.g., anxiety, attention problems, and aggression) (Jaffee et al., 2002; Caspi et al., 2014; Bartels et al., 2018; Whipp et al., 2021a), and these pathologies are also correlated genetically (Cross-Disorder Group of the Psychiatric Genomics Consortium Electronic address, 2019; Sprooten et al., 2022). Thus, it is also worth investigating biomarkers at a broader level of mental health, for example, the p factor. The p factor aggregates two underlying psychopathological processes: internalizing problems (e.g., anxiety and depression) and externalizing behavior (e.g., aggression and hyperactivity) into a generalized dimension (Caspi et al., 2014; Sprooten et al., 2022). Caspi et al. (2014) suggested that it may be difficult to identify biomarkers that are unique to specific psychiatric disorders, and that there may be biomarkers that are indicated at the p factor level. Thus, it would also be worth looking for clues about depression at this more aggregate level.

In this study, we therefore aimed to use a panel of low–molecular weight metabolites collected in young adulthood to investigate associations between biomarkers and depressive symptoms, as well as the p factor, in adolescence and young adulthood. In the p factor investigation, we also draw on findings from our previous study in the same dataset where we identified an association between ketone body 3-hydroxybutyrate and aggressive behavior in adolescence and young adulthood (Whipp et al., 2021b). This study is also a part of the Equal-Life consortium—-a consortium in the European Human Exposome Network—-which aims to investigate the effect of the internal (e.g., biomarkers) and external (e.g., physical environment and socioeconomic indicators) exposome on mental health in children, from conception to young adulthood (van Kamp et al., 2022). This analysis contributes to their investigation of the internal exposome.

## Materials and methods

### Cohort

This investigation used the FinnTwin12 cohort, which is an ongoing longitudinal population-based sample of Finnish twins born in 1983–1987 to investigate behavioral development and habits (Kaprio, 2006; Rose et al., 2019). Twins and their families were identified using the Finnish Central Population Registry, and the main full-sample waves of questionnaire collection occurred at ages 11/12, 14, 17, and 22 years. The baseline response rate was 87% (*N* = 5,600 twins) and has remained high throughout (response rate range: 85–90%). At age 14 years, a subset of the twins (from 1,035 families) was created and more intensively studied [including semi-structured psychiatric interviews and additional questionnaires (age 14 and 22 years), as well as blood plasma samples (age 22 years)]. The “age 22” assessment wave involved 1,347 twin individuals (mean age = 22.4 years, SD = 0.70; response rate 73.0%), with 779 individuals attending in-person assessments and thus venous blood plasma samples could be collected. The remainder were assessed by telephone interviews, and questionnaires returned by mail.

The blood samples were collected after overnight fasting, which involved abstaining from alcohol and tobacco since the night before sampling. Plasma was immediately extracted and stored at −80°C. The samples were processed in one batch in the autumn of 2010 using the Nightingale (formerly, Brainshake) automated high-throughput ^1^H nuclear magnetic resonance spectroscopy (NMR) metabolomics platform (Soininen et al., 2015; Bogl et al., 2016; Rose et al., 2019). The metabolites from the panel included were amino acids (alanine, glutamine, histidine, isoleucine, leucine, phenylalanine, tyrosine, and valine) and ketone bodies (acetate, acetoacetate, and 3-hydroxybutyrate). All metabolite data were available in units mmol/l. Pregnant women (*n* = 53) and one individual using cholesterol medication were excluded, yielding a final sample size of 725 twin individuals in this study.

Ethical approval for all data collection waves was obtained from the ethical committee of the Helsinki and Uusimaa University Hospital District and the Institutional Review Board of Indiana University. All data collection and sampling protocols were performed in compliance with the ethical guidelines. Parents provided consent for the twins aged 12 and 14 years, while twins aged 17 and 22 years provided written consent themselves for sample collection.

### Depression and p factor measures

In the FinnTwin12 cohort, behavioral and emotional development were measured at all waves. The modified Multidimensional Peer Nomination Inventory (MPNI) was used at ages 12, 14, and 17 years and was collected from parents (for age 12 years), teachers (for age 12 and 14 years), children themselves (for age 14 and 17 years), and the child’s co-twin (for age 14 and 17 years) (Pulkkinen et al., 1999). The MPNI is a 37-item questionnaire producing multiple subscales, including aggression (six items), depression (five items), hyperactivity/impulsivity (seven items), inattention (four items), and social anxiety (two items). Each item on the MPNI has four response choices (from “not observed in child” to “clearly observed”). Response choices are scored 0–3, and subscales are formed by taking the mean of all items in the subscale (no missing values were allowed).

An MPNI p factor variable was created by combining all the behavioral and emotional problem subscales together into a sum score, with no missing items allowed. A composite “combined” p factor score was also created by taking the mean of parent (12), teacher (12), and self (14) p factor values because we know that ratings from different raters are not highly correlated (Achenbach et al., 1987; Whipp et al., 2019, 2021a). The combined p factor is used in initial analyses to reduce issues of multiple testing, as was applied in our previous aggression biomarker study (Whipp et al., 2021b).

In addition, the FinnTwin12 study also collected self-rated depressive symptoms using the General Behavior Inventory (GBI) questionnaire at age 17 and 22 (Depue et al., 1981; Depue, 1987). The GBI is a 73-item questionnaire with multiple subscales characterizing mood-related behaviors, but a shorter 10-item version is used in FinnTwin12 to capture depressive symptoms. This shorter version has been successfully used previously (Edwards et al., 2011; Ranjit et al., 2019).

Each question in the 10-item GBI for depressive symptoms has four response choices (from “never” to “very often”). The response choices are scored 0–3, and a total sum score was created, with one missing value allowed.

To validate the GBI at age 22 years, we compared it with the Diagnostic and Statistical Manual of Mental Disorders (DSM-IV) diagnosis of major depressive disorder (MDD) assessed by the semi-structured psychiatric interview at age 22 years [Semi-Structured Assessment for the Genetics of Alcoholism (SSAGA)] (Bucholz et al., 1994). In a logistic regression analysis, the GBI age 22 score strongly predicted MDD, with the area under the receiver operating characteristic curve (AUC) of 0.8318.

### Covariates

Covariates included were available at the time of the plasma sample collection and were selected due to their possible association with both depression and biomarker levels. These included sex, body mass index (BMI), metabolic equivalent of task (MET), smoking status, alcohol frequency, and self-rated general health. BMI was used as a continuous measure (mean: 23.3, SD: 3.94) and calculated as kg/m^2^. MET hours/day were calculated from structured questions regarding intensity, frequency, and duration of activities (Kujala et al., 2019) to measure leisure time physical activity, which was used as a continuous measure. Current smoking status was categorized into current, former, and never/experimenter. Alcohol consumption frequency was categorized into none, once a month or less, 2–4 times per month, 2–3 times per week, and four or more times per week. Self-rated general health was used as a proxy for somatic health problems (e.g., diabetes) and was dichotomized into poor health (“poor” or “fair”) and good health (“good” or “very good”).

In addition, for a sensitivity analysis, we also considered anti-depressant use in the last 30 days as it might affect the biomarker levels and the depressive symptom score at age 22 years [GBI (22)]. From the SSAGA at age 22 years (Bucholz et al., 1994), we obtained all the participants who said they had used anti-depressant medication within the last 30 days (*n* = 34) (the interview was on the same day as the blood sample). Anti-depressant medication use in the last 30 days was then a yes/no binary.

### Analysis

For both depressive symptom (MPNI and GBI) and p factor (MPNI) scores, means and standard deviations (SD), as well as ranges, were computed for the different ages and raters. Correlations were computed between untransformed metabolites, and between metabolites and mental health measures (depressive symptom and p factor “combined” scores), as well as between depressive symptom scores and between p factor scores. Due to all metabolites having non-normal distributions, they were then rank-transformed (generally the best solution for most of the metabolites) for utilization in modeling.

**FIGURE 1:**
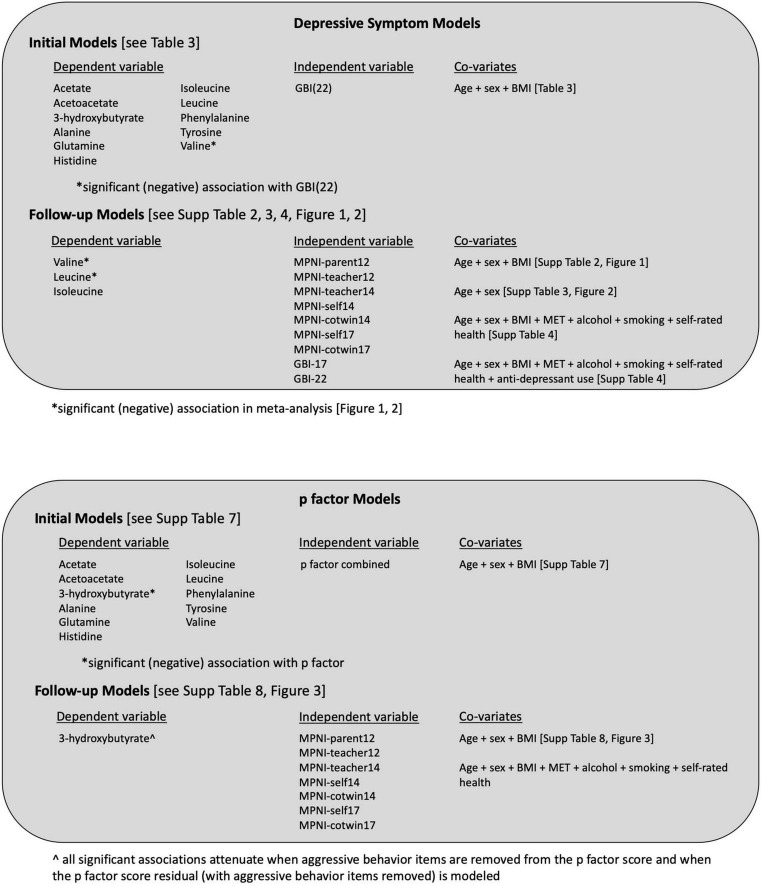
Overview of models and results from all depressive symptoms and p factor model analyses. BMI, body mass index; GBI, General Behavior Inventory; MET, Metabolic Equivalent of Task; MPNI, Multidimensional Peer Nomination Inventory.

For depressive symptom analyses, initial linear regression models included the metabolite (rank-transformed) as the dependent variable and GBI self-rating (age 22) score as the independent variable, adjusted for age, sex, BMI, and familial relatedness. Beta coefficients and 95% confidence intervals (CIs) were reported, and those reaching significance (*p* < 0.05) were further investigated. Additional analyses used depressive symptom scores captured before the metabolite data (at age 22); thus, eight separate models per metabolite were run using GBI self-rating (age 17), MPNI self-rating (age 17), MPNI co-twin rating (age 17), MPNI self-rating (age 14), MPNI co-twin rating (age 14), MPNI teacher rating (age 14), MPNI teacher rating (age 12), or MPNI parent rating (age 12) as the main independent variable, adjusted for age, sex, BMI, and familial relatedness. Previous investigations have shown that different raters and ages can provide unique contributions to associations with mental health ratings (Whipp et al., 2019, 2021a). The depressive symptom scores were standardized with mean 0, SD 1 for comparability. In addition, a meta-analysis was performed to summarize across all nine models run per biomarker. Furthermore, the models were fully adjusted to also include MET, smoking status, alcohol frequency, and self-rated general health, and a meta-analysis was run for these. Sex interactions between sex and depressive symptom scores were also tested. Finally, two sensitivity analyses were run. First, BCAA depressive symptom models were run, where only age, sex, and familial relatedness were covariates (BMI was excluded) in order to see if BMI was driving any of the BCAA associations with depressive symptoms, and a meta-analysis was also run on these. Lastly, we added anti-depressant use in the last 30 days to the fully adjusted models with standardized GBI (22) score as the main independent variable, as well as ran the fully adjusted models, excluding all those who used anti-depressant medication in the last 30 days. An overview of the models analyzed can be seen in Figure 1.

For the p factor analyses, initial linear regression models included the metabolite (rank-transformed) as the dependent variable and p factor “combined” score as the independent variable, adjusted for age, sex, BMI, and familial relatedness. Beta coefficients and 95% CIs were reported, and those reaching significance (*p* < 0.05) were further investigated using the seven separate MPNI ratings (standardized with mean 0, SD 1), and a meta-analysis was run as well. In addition, fully adjusted models were run with addition covariates: MET, smoking status, alcohol frequency, and self-rated general health. Sex interactions between sex and p factor score were also tested. Finally, linear regression models were run with the aggression subscale items removed from the p factor score calculation (as well as removing the aggression residual from the p factor score), to see if our previous aggression biomarker analysis finding (Whipp et al., 2021b) was driving any p factor biomarker findings. An overview of the models analyzed can be seen in Figure 1.

## Results

### Depressive symptoms

The mean, SD, and range of depressive symptom scores across the nine ratings are presented in Table 1.

**TABLE 1:** Mean, standard deviation (SD), and range of depressive symptom scores (MPNI and GBI) by ratings.

Depressive symptom variable	N	Mean	SD	Min.	Max.
MPNI parent (12)	698	0.75	0.43	0	2.4
MPNI teacher (12)	708	0.64	0.52	0	2.8
MPNI teacher (14)	567	0.53	0.46	0	2.4
MPNI self (14)	696	0.66	0.41	0	2.4
MPNI co-twin (14)	699	0.61	0.41	0	2.2
MPNI self (17)	658	0.73	0.67	0	3
MPNI co-twin (17)	653	0.67	0.63	0	3
GBI self (17)	658	4.93	4.76	0	30
GBI self (22)	718	4.47	4.59	0	29

GBI, General Behavior Inventory; MPNI, Multidimensional Peer Nomination Inventory.

Correlations between the GBI (22) and metabolites were low and often negative, from −0.22 to 0.03 (Table 2). Correlations between metabolites were as expected. Significant correlations (*p* < 0.05) with depressive symptoms occurred for acetate, isoleucine, leucine, tyrosine, and valine. In addition, correlations between the nine different depressive symptom scores were low to moderate (r range: 0.02–0.49; Supplementary Table 1).

**TABLE 2:** Spearman correlations between untransformed metabolites and GBI (22) variables (*n* = 701).

	Acetate	Acetoacetate	3-hydroxybutyrate	Alanine	Glutamine	Histidine	Isoleucine	Leucine	Phenylalanine	Tyrosine	Valine
Acetoacetate	**0.13**										
3-hydroxybutyrate	**0.17**	**0.73**									
Alanine	**−0.15**	**−0.36**	**−0.35**								
Glutamine	**0.33**	−0.06	−0.06	**0.08**							
Histidine	0.07	−0.03	**−0.08**	**0.19**	0.01						
Isoleucine	**0.09**	**0.21**	−0.04	**0.21**	**0.26**	**0.09**					
Leucine	**0.08**	**0.13**	−0.02	**0.11**	**0.16**	−0.001	**0.71**				
Phenylalanine	**−0.09**	−0.03	−0.02	**0.18**	**−0.32**	**0.29**	**0.15**	**0.36**			
Tyrosine	**0.15**	**−0.12**	**−0.21**	**0.40**	**0.38**	**0.18**	**0.42**	**0.30**	**0.16**		
Valine	**0.20**	**0.19**	0.01	**0.08**	**0.35**	0.05	**0.68**	**0.61**	0.02	**0.53**	
GBI (22)	**−0.08**	−0.03	0.005	0.02	−0.05	0.009	**−0.13**	**−0.15**	0.03	**−0.11**	**−0.22**

GBI, General Behavior Inventory. *p* < 0.05 correlations in bold.

Initial regression models with the metabolite as the dependent factor and GBI depressive symptom score (age 22) as the independent factor, adjusted for age, sex, BMI, and familial relatedness are presented in Table 3. The depressive symptom score was only significantly associated with valine, accounting for 3.6% of the variance in depressive symptoms. Valine was the only biomarker that reached a nominal *p* < 0.05, adjusted for multiple testing for nine independent variables obtained from matrix spectral decomposition (matSpD),^1^ which estimated the equivalent number of independent variables in our correlation matrix of 11 metabolites.

**TABLE 3:** Regression models for metabolites as the dependent variable and GBI depressive symptom (age 22) score as the main independent variable.

Biomarker^a^	Co-variates	N	Unstandardized GBI (22) beta coeff (95% CI)	R-squared GBI (22) only^b^	R-squared full^c^
Acetate	age, sex*, BMI	716	−1.58 (−5.3, 2.2)	0.4%	4.9%
Acetoacetate	age*, sex*, BMI*	715	1.7 (−2.3, 5.7)	0.01%	8.2%
3-hydroxybutyrate	age, sex, BMI*	705	−0.2 (−4.3, 4.0)	0%	3.0%
Alanine	age, sex, BMI*	716	2.5 (−1.0, 6.0)	0.1%	2.1%
Glutamine	age*, sex*, BMI	716	1.8 (−1.2, 4.8)	0.05%	30.7%
Histidine	age, sex, BMI	716	0.6 (−3.3, 4.4)	0.06%	1.5%
Isoleucine	age, sex*, BMI*	714	1.3 (−1.7, 4.3)	0.3%	28.4%
Leucine	age*, sex*, BMI*	714	−2.4 (−5.6, 0.8)	1.2%	17.4%
Phenylalanine	age, sex*, BMI*	716	−0.2 (−3.3, 2.9)	0%	15.9%
Tyrosine	age, sex*, BMI*	710	−1.6 (−5.4, 2.1)	0.9%	17.4%
Valine	age, sex*, BMI*	710	−4.8 (−7.8, −1.7)	3.6%	40.7%

BMI, body mass index; CI, confidence interval; GBI, General Behavior Inventory.

*This co-variate also significant (*p* < 0.05).

^a^Biomarkers are all rank-transformed.

^b^R-squared here represents the % variation explained from a model with only GBI (22) as the independent variable (no age, sex, or BMI included).

^c^R-squared here represents the % variation explained from a model with GBI (22), age, sex, and BMI in the model.

**FIGURE 2:**
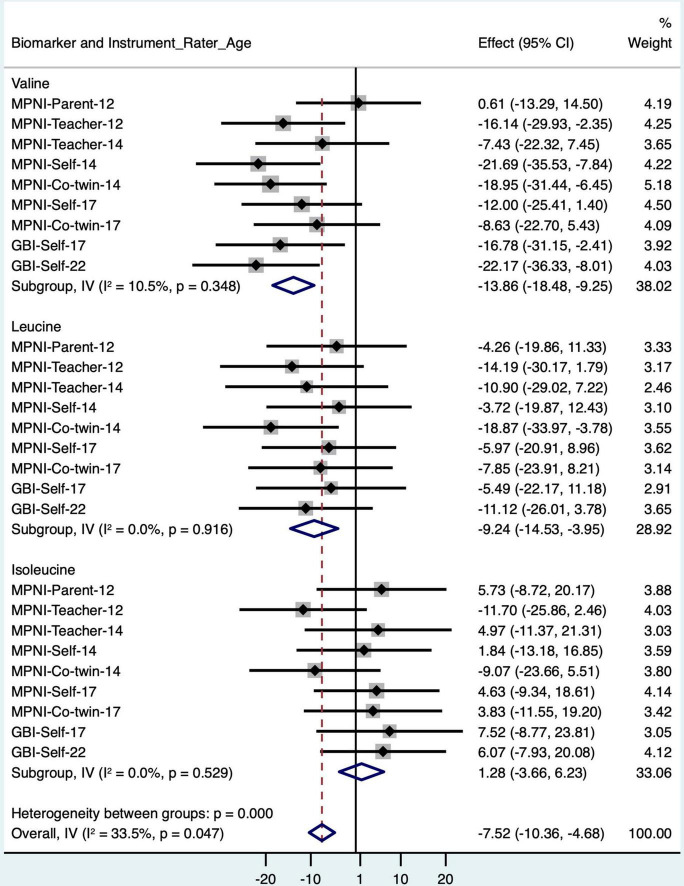
Forest plot of BCAA models with standardized depressive symptom scores from different instruments, raters, and ages (with age, sex, and BMI as covariates), and meta-analysis of all models for each BCAA biomarker. BCAA, branched-chain amino acid; CI, confidence interval; GBI, General Behavior Inventory; MPNI, Multidimensional Peer Nomination Inventory.

Valine is highly correlated with the other branched-chain amino acids (BCAA; correlations between the BCAAs: 0.61–0.71), and BCAAs have been shown to be associated with depression in a few previous studies (Baranyi et al., 2016; Bot et al., 2020; Koochakpoor et al., 2021). Therefore, we looked closer at valine and the other BCAAs (isoleucine and leucine) regarding associations with depressive symptoms at earlier time points (ages 12, 14, and 17 years) to see if there is continuity in the association across ages (Figure 2 and Supplementary Table 2). According to the meta-analysis (Figure 2), valine showed an overall negative association with depressive symptoms [meta-analysis beta (valine) = −13.86, 95% CI (−18.48 to −9.25)]. Leucine also showed an overall negative association [meta-analysis beta (leucine) = −9.24, 95% CI (−14.53 to −3.95)]. Isoleucine, however, was not significantly associated with depressive symptoms [meta-analysis beta (isoleucine) = −0.95, 95% CI (−6.00 to 4.11)]. In fully adjusted models, including meta-analysis, results for valine, leucine, and isoleucine remained similar [meta-analysis beta (valine) = −11.20, 95% CI (−15.80 to −6.59); meta-analysis beta (leucine) = −9.86, 95% CI (−15.27 to −4.46); meta-analysis beta (isoleucine) = 0.81, 95% CI (−4.26 to 5.88)]. No sex–depressive symptoms interactions were detected.

**FIGURE 3:**
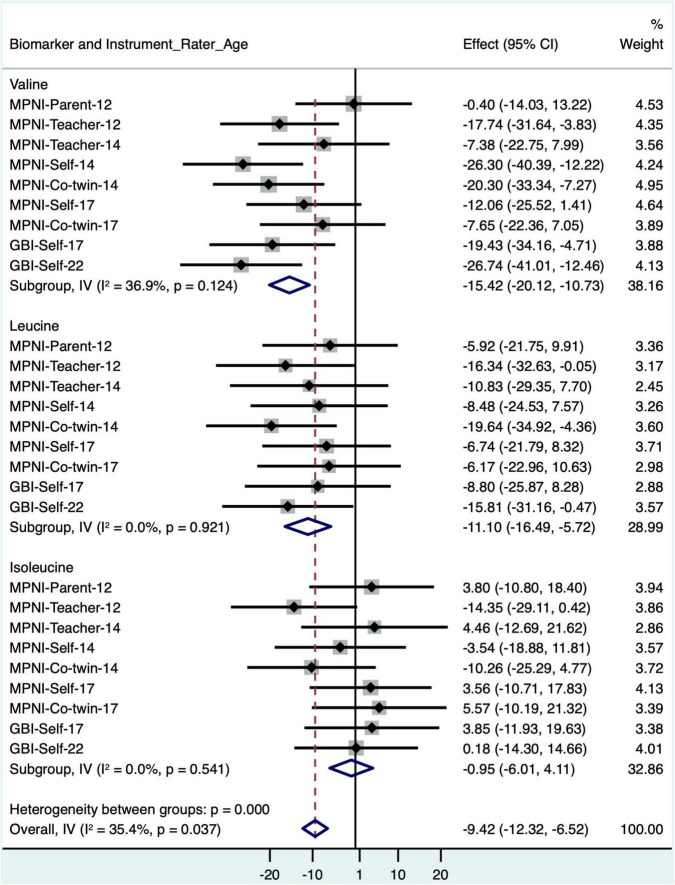
Forest plot of BCAA models with standardized depressive symptom scores from different instruments, raters, and ages (with age and sex as covariates, BMI removed), and meta-analysis of all models for each BCAA biomarker. BCAA, branched-chain amino acid; CI, confidence interval; GBI, General Behavior Inventory; MPNI, Multidimensional Peer Nomination Inventory.

In models where BMI was removed, valine and leucine remained significantly negatively associated with depressive symptoms, while isoleucine remained non-significantly associated with depressive symptoms (Figure 3 and Supplementary Table 3).

In sensitivity models where anti-depressant medication use in the last 30 days was factored in, valine and isoleucine remained significantly negatively associated with depressive symptoms, both in models where anti-depressants were adjusted for and where those participants taking anti-depressants (*n* = 34) were removed from analyses (Supplementary Table 4). The lack of association of isoleucine with depressive symptoms did not change in these anti-depressant sensitivity models.

### p factor

The mean, SD, and range of p factor scores across the seven ratings (and a “combined” p factor variable) are presented in Supplementary Table 5.

Correlations between the p factor “combined” score and metabolites were low, ranging −0.11 to 0.10. Significant correlations (*p* < 0.05) occurred only for 3-hydroxybutyrate, glutamine, isoleucine, and phenylalanine. In addition, correlations between the seven p factor scores from different raters and ages were low to moderate (r range: 0.10–0.53; Supplementary Table 6). Initial regression models with the metabolite as the dependent factor and p factor “combined” score as the independent factor, adjusted for age, sex, BMI, and familial relatedness are presented in Supplementary Table 7. The p factor was only significantly associated with 3-hydroxybutyrate.

**FIGURE 4:**
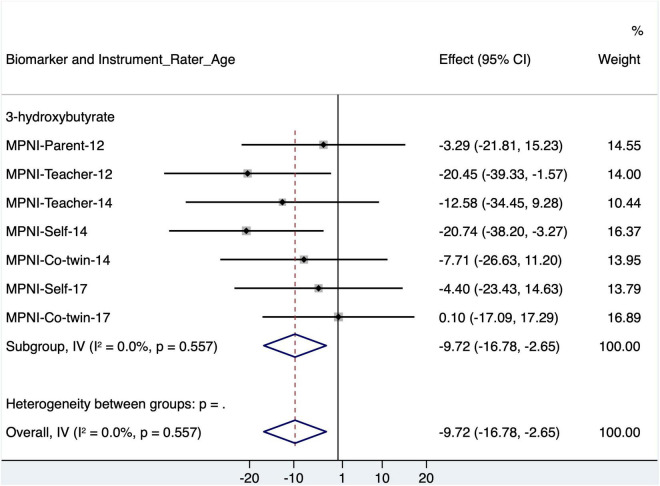
Forest plot of 3-hydroxybutyrate models with standardized p factor scores from different raters and ages (with age, sex, and BMI as covariates), as well as meta-analysis of all models. CI, confidence interval; MPNI, Multidimensional Peer Nomination Inventory.

Looking closer at 3-hydroxybutyrate with the p factor score, separate models were run using all seven ratings with p factor scores (Figure 4 and Supplementary Table 8). For 3-hydroxybutyrate, all ratings [except co-twin (17)] showed the negative association, but only teacher (12) and self (14) were significant [similar to the association pattern in Whipp et al. (2021b)]. In fully adjusted models (age, sex, BMI, MET, smoking status, alcohol frequency, and self-rated general health), the 3-hydroxybutyrate and p factor association remained for both teacher (12) (*p* = 0.041) and self (14) (*p* = 0.018). Of these, only the teacher (12) p factor association with 3-hydroxybutyrate suggests a p factor–sex interaction (*p* = 0.046).

In models where the aggressive behavior subscale is removed from the p factor score (to see if the influence of the known 3-hydroxybutyrate–aggression association was driving the 3-hydroxybutyrate–p factor association), the 3-hydroxybutyrate models are no longer significant. In a final sensitivity analysis where the p factor score (with aggression scale items removed) residual was modeled (i.e., even the effect of aggression on other scales in the p factor is removed), all 3-hydroxybutyrate models remained non-significant.

## Discussion

In this large population-based cohort of adolescent and young adult Finnish twins, a negative association between BCAAs valine and leucine was observed with depressive symptoms (Figure 1). In the initial investigation with the panel of 11 low–molecular weight metabolites, GBI–depressive symptom scores at age 22 years were negatively associated with valine (higher levels of depressive symptoms were associated with lower levels of valine). Considering all three BCAAs more closely—-using multiple ratings of depressive symptoms from different ages and raters as well as different model adjustments—-the negative associations with valine and leucine with depressive symptoms were a consistent and often significant trend across raters and ages. In a meta-analysis across the different depressive symptom measures, ages, and raters, the overall associations of both valine and leucine with depressive symptoms were significant. Despite the expected sex difference in depressive symptoms, no sex interactions were found in the associations. Thus, although female participants had higher levels of depressive symptoms than male participants, which is also well established in the literature (Otte et al., 2016), the association between the biomarker level and depressive symptoms is not different for female and male participants. In addition, looking for metabolite associations with the broader mental health dimension, the p factor did not yield any new findings, and the negative valine association (seen with depression) was no longer significant, suggesting it is still important to investigate biomarker associations within a specific psychopathology phenotype (Figure 1).

The association found in this study between BCAAs and depression has been found in a few previous cross-sectional adult samples; however, this appears to be the first time the association was demonstrated in an adolescent/young adult sample. A small, clinical, cross-sectional study of Austrian adults (the majority were male patients) found an association between low BCAAs and higher depression (Baranyi et al., 2016). In Koochakpoor et al. (2021), an analysis using a large Iranian adult cross-sectional dataset with dietary BCAA intake information found that individuals with diets high in BCAAs (and valine in particular) had lower levels of depression. However, a large study investigating metabolomic associations with depression in nine Dutch cohorts (mean age range 40.4–64.8 years) did not find a significant valine–depression association, but they did identify a positive association of isoleucine with depression (Bot et al., 2020). Our findings also note a similar (although weak and non-significant) positive trend between isoleucine and depressive symptoms. One possible reason our results do not fully align with the Dutch study could be the age differences of the samples. Our cross-sectional association at age 22 years between valine and depressive symptoms appears to go back as far as age 12 years, so there is stability in the association as well. Although it is unclear which direction the association is acting (metabolite levels affect future depressive symptoms, or depressive symptoms affect future metabolite levels), it could be that valine and leucine are important earlier biomarkers of depression, and isoleucine becomes notable in later ages.

Regarding potential biological mechanisms for the BCAA–depression associations, the energy metabolism pathways seem a plausible explanation. This pathway is one of the main categories of mechanisms of depression (Strawbridge et al., 2017; Zwolinska et al., 2021), and there are known links between BCAAs and, for example, obesity (Pietilainen et al., 2008), BMI (Bogl et al., 2016), and diabetes (Wang et al., 2011). These conditions—-obesity, high BMI, and diabetes—-are also associated with depression (Pan et al., 2012a,b; Milaneschi et al., 2019). In our study, when modeling the biomarker–depression associations, we tested models with age and sex only, as well as age, sex, and BMI, and then fully adjusted models. For valine and leucine, the associations remained across all model adjustments, suggesting the association between valine and leucine with depression is independent of the known BMI–BCAA relationship. Baranyi et al. (2016), who identified a BCAA–depression link, suggested a deficiency in BCAAs, leading to a reduction in the mammalian target of rapamycin (mTor) activity, which would result in lower energy metabolism and depressive symptoms. Several depression medications (e.g., ketamine, fluoxetine, and methylphenidate) seem to have an effect on mTor as well (Warren et al., 2011; Abelaira et al., 2014). In our sensitivity analyses, anti-depressant medication use in the last 30 days, which was infrequent in our sample (*n* = 34), did not appear to have an effect on the association between BCAAs and depressive symptom score.

mTor has also been suggested as a biological mechanism in the BCAA–obesity relationship (Pietilainen et al., 2008), although our valine and leucine associations with depression appear to be generally independent of this association. However, Martins-de-Souza (2014) identified a protein–metabolite interactome relationship of phosphatidylinositol 3-kinase with mTor that has a role in depression therapeutics in immunity and neurotransmitter biological mechanisms. Thus, while BCAAs may be affecting mTor activity in depression, the biology is still far from clear, but important to further explore and elucidate.

Lastly, this study, as well as the Equal-Life consortium (of which this study is a part), aimed to look beyond depression, at metabolite associations with the broader, overall mental health concept, the p factor. We had previously found a negative association with an externalizing psychopathology (aggressive behavior) and the ketone body 3-hydroxybutyrate (Whipp et al., 2021b), and here studied an internalizing psychopathology (depression). We also aimed to investigate whether there were further insights to be gained with metabolite associations at the p factor level (which combines externalizing and internalizing psychopathologies). In our investigation, we did not see any significant associations between metabolites and the p factor, except 3-hydroxybutyrate, which was attributed to the association with aggression. To our knowledge, there are no other studies of metabolite biomarkers of the p factor. Although Caspi et al. (2014) suggested that there are likely biomarkers that are associated with the p factor, ones that do not discriminate between psychiatric disorders, in this study, we did not find evidence of that phenomenon, based on our restricted set of low–molecular weight metabolites. We have identified a biomarker unique to an internalizing problem (depression) and a biomarker unique to an externalizing problem (aggression; Whipp et al., 2021b). While there may be common biological pathways to explain some aspects of broad mental health problems, it is still likely that there are unique aspects that clarify single, or closely related, biological pathways for specific mental health problems. Further work also needs to explore whether specific environmental exposures affect the BCAA–depression associations. Collectively known as the exposome, the relevant agents may be physico-chemical (such as air pollution), social (such as childhood family relationships and childhood traumatic experiences), or biological (such as diet or microbiome).

This study has many strengths, including a large population-based sample, biomarker and depression measures at the same time point as well as depression measures longitudinally, and multiple raters and instruments of depression. In addition, there are some limitations to consider. First, we only had one time point for biomarker data. Although we observed stability in the depression–metabolite associations over time, it would still be important to have multiple time points with both depression measures and metabolite samples. This would be especially important in identifying the direction of the association.

In summary, we identified a negative association between BCAA metabolites valine and leucine and depressive symptoms. These associations were consistent and robust across time points and raters and model adjustments. This association is shown for the first time in a young population-based cohort, with a few studies having also shown BCAA–depression associations in older adult cohorts. In addition, investigations did not find any biomarker associations with the broader mental health measure—the p factor. Further exploration is necessary to investigate the temporality of the metabolite levels with depression onset and to better characterize which biological mechanisms are involved in the association in order to improve our understanding of depression and its prognosis and treatment.

## Data availability statement

The data analyzed in this study is subject to the following licenses/restrictions: The FT12 data is not publicly available due to the restrictions of informed consent. Requests to access these datasets should be directed to the Institute for Molecular Medicine Finland (FIMM) Data Access Committee (DAC) (fimmdac@helsinki.fi) for authorized researchers who have IRB/ethics approval and an institutionally approved study plan. To ensure the protection of privacy and compliance with national data protection legislation, a data use/transfer agreement is needed, the content and specific clauses of which will depend on the nature of the requested data.

## Ethics statement

The studies involving human participants were reviewed and approved by the Helsinki and Uusimaa University Hospital District and Indiana University’s Institutional Review Board. Written informed consent to participate in this study was provided by the participants’ legal guardian/next of kin (ages 12 and 14) and the participants themselves (ages 17 and 22).

## Author contributions

AW helped design the study, analyzed the data, wrote the manuscript, and managed the drafts. MH-G helped in the study design planning and critically reviewed the manuscript drafts. KP helped in interpreting the findings and critically reviewed the manuscript drafts. IK helped in designing the study and critically reviewed the manuscript drafts. JK helped in designing the study, interpreting the results, and critically reviewed the manuscript drafts. All authors contributed to the article and approved the submitted version.

## Footnotes

1
https://web.archive.org/web/20210310030115/https://neurogenetics.qimrberghofer.edu.au/matSpD/
