# Supplementary material for: Branched-chain amino acids linked to depression in young adults
Source: Front Neurosci. 2022 Sep 30;16:935858. doi: 10.3389/fnins.2022.935858 (PMC9561956; doi:10.3389/fnins.2022.935858)
Supplement: Supplementary file 1 [file Data_Sheet_1.docx]

Supplemental Table 1. Spearman correlations between depressive symptom scores (p<0.05 correlations in bold)(n=489)

|  | MPNI Teacher(12) | MPNI Teacher(14) | MPNI Self(14) | MPNI Co-twin(14) | MPNI Self(17) | MPNI Co-twin(17) | GBI Self(17) | GBI Self(22) |
| --- | --- | --- | --- | --- | --- | --- | --- | --- |
| MPNI Parent(12) | **0.26** | **0.24** | **0.15** | **0.15** | **0.13** | **0.09** | 0.06 | 0.07 |
| MPNI Teacher(12) |  | **0.33** | **0.11** | 0.16 | 0.05 | 0.05 | 0.03 | 0.08 |
| MPNI Teacher(14) |  |  | **0.11** | **0.18** | **0.15** | 0.09 | 0.02 | **0.12** |
| MPNI Self(14) |  |  |  | **0.31** | **0.38** | **0.22** | **0.38** | **0.29** |
| MPNI Co-twin(14) |  |  |  |  | **0.18** | **0.33** | **0.20** | **0.19** |
| MPNI Self(17) |  |  |  |  |  | **0.38** | **0.37** | **0.31** |
| MPNI Co-twin(17) |  |  |  |  |  |  | **0.20** | **0.20** |
| GBI Self(17) |  |  |  |  |  |  |  | **0.49** |

Abbreviations: GBI=General Behavior Inventory; MPNI=Multidimensional Peer Nomination Inventory

Supplemental Table 2. Regression models for branched-chain amino acids (isoleucine, leucine, valine) as dependent variables and standardized depressive symptom scores from different raters as the independent variable

| **Biomarker^a^** | **Instrument-Rater-Age** | **Co-variates** | **N** | **Standardized**  **Depressive Symptom Score Beta Coeff**  **(95% CI)** | **R-squared^b^** |
| --- | --- | --- | --- | --- | --- |
| isoleucine | MPNI-Parent-12 | age, sex*, BMI* | 690 | 5.73 (-8.72, 20.17) | 28.4% |
|  | MPNI-Teacher-12 | age, sex*, BMI* | 700 | -11.70 (-25.86, 2.46) | 28.2% |
|  | MPNI-Teacher-14 | age, sex*, BMI* | 561 | 4.97 (-11.37, 21.31) | 32.0% |
|  | MPNI-Self-14 | age, sex*, BMI* | 688 | 1.84 (-13.18, 16.85) | 28.5% |
|  | MPNI-Co-twin-14 | age, sex*, BMI* | 692 | -9.07 (-23.66, 5.51) | 28.9% |
|  | MPNI-Self-17 | age, sex*, BMI* | 652 | 4.63 (-9.34, 18.61) | 29.9% |
|  | MPNI-Co-twin-17 | age, sex*, BMI* | 646 | 3.83 (-11.55, 19.20) | 29.7% |
|  | GBI-Self-17 | age, sex*, BMI* | 652 | 7.52 (-8.77, 23.81) | 29.9% |
|  | GBI-Self-22 | age, sex*, BMI* | 714 | 6.07 (-7.93, 20.08) | 28.4% |
|  |  |  |  |  |  |
| leucine | MPNI-Parent-12 | age*, sex*, BMI* | 690 | -4.26 (-19.86, 11.33) | 17.1% |
|  | MPNI-Teacher-12 | age*, sex*, BMI* | 700 | -14.19 (-30.17, 1.79) | 17.3% |
|  | MPNI-Teacher-14 | age*, sex*, BMI* | 561 | -10.90 (-29.02, 7.22) | 18.7% |
|  | MPNI-Self-14 | age*, sex*, BMI* | 688 | -3.72 (-19.87, 12.43) | 17.8% |
|  | MPNI-Co-twin-14 | age*, sex*, BMI* | 692 | -18.87 (-33.97, -3.78) | 19.0% |
|  | MPNI-Self-17 | age*, sex*, BMI* | 652 | -5.97 (-20.91, 8.97) | 19.2% |
|  | MPNI-Co-twin-17 | age*, sex*, BMI* | 646 | -7.85 (-23.91, 8.21) | 19.2% |
|  | GBI-Self-17 | age*, sex*, BMI* | 652 | -5.49 (-22.17, 11.18) | 19.0% |
|  | GBI-Self-22 | age*, sex*, BMI* | 714 | -11.12 (-26.01, 3.78) | 17.4% |
|  |  |  |  |  |  |
| valine | MPNI-Parent-12 | age, sex*, BMI* | 686 | 0.61 (-13.29, 14.50) | 39.6% |
|  | MPNI-Teacher-12 | age, sex*, BMI* | 696 | -16.14 (-29.93, -2.35) | 39.7% |
|  | MPNI-Teacher-14 | age, sex*, BMI* | 557 | -7.43 (-22.32, 7.45) | 39.2% |
|  | MPNI-Self-14 | age, sex*, BMI* | 685 | -21.69 (-35.53, -7.85) | 41.1% |
|  | MPNI-Co-twin-14 | age, sex*, BMI* | 688 | -18.95 (-31.44, -6.45) | 41.6% |
|  | MPNI-Self-17 | age, sex*, BMI* | 648 | -12.00 (-25.41, 1.40) | 39.7% |
|  | MPNI-Co-twin-17 | age, sex*, BMI* | 642 | -8.63 (-22.71, 5.44) | 39.6% |
|  | GBI-Self-17 | age, sex*, BMI* | 648 | -16.78 (-31.15, -2.41) | 39.9% |
|  | GBI-Self-22 | age, sex*, BMI* | 710 | -22.17 (-36.33, -8.01) | 40.7% |

Abbreviations: GBI=General Behavior Inventory; MPNI=Multidimensional Peer Nomination Inventory

*this co-variate is also significant (p<0.05)

^a^biomarkers are all rank-transformed

^b^R-squared represents the variation explained from all variables in the model together

Supplemental Table 3. Linear regression models for branched-chain amino acids (isoleucine, leucine, valine) as the dependent variable and standardized depressive symptom scores from different raters as the independent variable with BMI removed as a covariate

| **Biomarker^a^** | **Instrument-Rater-Age** | **Co-variates** | **N** | **Standardized**  **Depressive Symptom Score Beta Coeff**  **(95% CI)** | **R-squared^b^** |
| --- | --- | --- | --- | --- | --- |
| isoleucine | MPNI-Parent-12 | age, sex* | 696 | 3.80 (-10.80, 18.40) | 22.6% |
|  | MPNI-Teacher-12 | age, sex* | 706 | -14.35 (-29.11, 0.42) | 22.5% |
|  | MPNI-Teacher-14 | age, sex* | 565 | 4.46 (-12.69, 21.62) | 26.6% |
|  | MPNI-Self-14 | age, sex* | 694 | -3.54 (-18.88, 11.81) | 22.7% |
|  | MPNI-Co-twin-14 | age, sex* | 697 | -10.26 (-25.29, 4.77) | 23.0% |
|  | MPNI-Self-17 | age, sex* | 656 | 3.56 (-10.71, 17.83) | 24.8% |
|  | MPNI-Co-twin-17 | age, sex* | 651 | 5.57 (-10.19, 21.32) | 24.3% |
|  | GBI-Self-17 | age, sex* | 656 | 3.85 (-11.93, 19.63) | 24.7% |
|  | GBI-Self-22 | age, sex* | 716 | 0.18 (-14.30, 14.66) | 23.1% |
|  |  |  |  |  |  |
| leucine | MPNI-Parent-12 | age*, sex* | 696 | -5.92 (-21.75, 9.91) | 12.8% |
|  | MPNI-Teacher-12 | age*, sex* | 706 | -16.34 (-32.63, -0.05) | 12.9% |
|  | MPNI-Teacher-14 | age*, sex* | 565 | -10.83 (-29.35, 7.70) | 14.4% |
|  | MPNI-Self-14 | age, sex* | 694 | -8.48 (-24.53, 7.57) | 13.2% |
|  | MPNI-Co-twin-14 | age*, sex* | 697 | -19.64 (-34.92, -4.36) | 14.2% |
|  | MPNI-Self-17 | age*, sex* | 656 | -6.74 (-21.79, 8.32) | 14.6% |
|  | MPNI-Co-twin-17 | age*, sex* | 651 | -6.17 (-22.96, 10.63) | 14.3% |
|  | GBI-Self-17 | age*, sex* | 656 | -8.80 (-25.87, 8.28) | 14.5% |
|  | GBI-Self-22 | age*, sex* | 716 | -15.81 (-31.16, -0.47) | 13.4% |
|  |  |  |  |  |  |
| valine | MPNI-Parent-12 | age, sex* | 692 | -0.40 (-14.03, 13.22) | 33.9% |
|  | MPNI-Teacher-12 | age, sex* | 702 | -17.74 (-31.64, -3.83) | 34.7% |
|  | MPNI-Teacher-14 | age, sex* | 561 | -7.38 (-22.75, 7.99) | 33.4% |
|  | MPNI-Self-14 | age, sex* | 691 | -26.30 (-40.39, -12.22) | 35.6% |
|  | MPNI-Co-twin-14 | age, sex* | 693 | -20.30 (-33.34, -7.27) | 36.1% |
|  | MPNI-Self-17 | age, sex | 652 | -12.06 (-25.52, 1.41) | 34.0% |
|  | MPNI-Co-twin-17 | age, sex* | 647 | -7.65 (-22.36, 7.05) | 33.4% |
|  | GBI-Self-17 | age, sex* | 652 | -19.43 (-34.16, -4.71) | 34.4% |
|  | GBI-Self-22 | age, sex* | 712 | -26.74 (-41.01, -12.46) | 35.5% |

Abbreviations: GBI=General Behavior Inventory; MPNI=Multidimensional Peer Nomination Inventory

*this co-variate is also significant (p<0.05)

^a^biomarkers are all rank-transformed

^b^R-squared represents the variation explained from all variables in the model together

Supplemental Table 4. Linear regression models for branched-chain amino acids (isoleucine, leucine, valine) as the dependent variable and standardized GBI(22) depressive symptom scores in a fully adjusted model, a fully adjusted model with anti-depressant use in last 30 days added, and a fully adjusted model excluding those who used anti-depressants in the last 30 days

| **Biomarker^a^** | **Instrument-Rater-Age** | **Co-variates** | **N** | **Standardized**  **Depression Score Beta Coeff**  **(95% CI)** | **R-squared^b^** |
| --- | --- | --- | --- | --- | --- |
| isoleucine | GBI-Self-22 | age, sex*, BMI*, MET, alcohol, smoking, self-rated health | 708 | 3.84 (-10.47, 18.15) | 28.3% |
|  | GBI-Self-22 | age, sex*, BMI*, MET, alcohol, smoking, self-rated health, anti-depressants* | 708 | -1.52 (-16.82, 13.78) | 28.7% |
|  | GBI-Self-22 | age, sex*, BMI*, MET, alcohol, smoking, self-rated health | 689^c^ | -5.03 (-20.97, 10.92) | 28.2% |
|  |  |  |  |  |  |
| leucine | GBI-Self-22 | age*, sex*, BMI*, MET, alcohol, smoking, self-rated health | 708 | -14.39 (-29.93, 1.15) | 18.0% |
|  | GBI-Self-22 | age*, sex*, BMI*, MET, alcohol, smoking, self-rated health, anti-depressants | 708 | -19.08 (-35.36, -2.79) | 18.3% |
|  |  | age*, sex*, BMI*, MET, alcohol, smoking, self-rated health | 689^c^ | -22.55 (-39.30, -5.79) | 18.3% |
|  |  |  |  |  |  |
| valine | GBI-Self-22 | age, sex*, BMI*, MET*, alcohol, smoking, self-rated health* | 705 | -18.90 (-32.92, -4.87) | 41.5% |
|  | GBI-Self-22 | age, sex*, BMI*, MET, alcohol, smoking, self-rated health*, anti-depressants | 705 | -17.50 (-32.33, -2.67) | 41.6% |
|  | GBI-Self-22 | age, sex*, BMI*, MET*, alcohol, smoking, self-rated health | 686^c^ | -18.47 (-34.01, -2.92) | 41.4% |

Abbreviations: GBI=General Behavior Inventory; MPNI=Multidimensional Peer Nomination Inventory

*this co-variate is also significant (p<0.05)

^a^biomarkers are all rank-transformed

^b^R-squared represents the variation explained from all variables in the model together

^c^Those who used anti-depressant medication in last 30 days are excluded from model

Supplemental Table 5. Mean, standard deviation (SD) and range of Multidimensional Peer Nomination Inventory p factor scores by ratings

| Variable | N | Mean | SD | Min | Max |
| --- | --- | --- | --- | --- | --- |
| Parent(12) | 692 | 16.3 | 8.0 | 0 | 49 |
| Teacher(12) | 692 | 15.4 | 11.4 | 0 | 61 |
| Teacher(14) | 556 | 11.1 | 9.3 | 0 | 50 |
| Self(14) | 686 | 16.4 | 6.8 | 2 | 44 |
| Co-twin(14) | 679 | 16.5 | 8.2 | 1 | 46 |
| Self(17) | 634 | 19.2 | 6.5 | 3 | 41 |
| Co-twin(17) | 630 | 19.1 | 7.8 | 3 | 46 |
| Combined* | 626 | 16.0 | 6.5 | 4 | 46.7 |

*“combined” variable was created by taking the mean of parent(12), teacher(12) and self(14) values

Supplemental Table 6. Spearman correlations between p factor scores from the Multidimensional Peer Nomination Inventory raters at ages 12, 14 and 17 (all correlations significant at p<0.05)(n=420)

|  | p factor Teacher(12) | p factor Teacher(14) | p factor Self(14) | p factor Co-twin(14) | p factor Self(17) | p factor Co-twin(17) |
| --- | --- | --- | --- | --- | --- | --- |
| p factor Parent(12) | 0.30 | 0.32 | 0.28 | 0.33 | 0.21 | 0.28 |
| p factor Teacher(12) |  | 0.40 | 0.22 | 0.30 | 0.10 | 0.20 |
| p factor Teacher(14) |  |  | 0.28 | 0.36 | 0.17 | 0.23 |
| p factor Self(14) |  |  |  | 0.42 | 0.47 | 0.37 |
| p factor Co-twin(14) |  |  |  |  | 0.28 | 0.53 |
| p factor Self(17) |  |  |  |  |  | 0.46 |

Supplemental Table 7. Linear regression models for metabolites as the dependent variable and Multidimensional Peer Nomination Inventory p factor “combined” score as the main independent variable, adjusted for age, sex, BMI and familial relatedness

| **Biomarker^a^** | **Co-variates** | **N** | **Unstandardized**  **p Factor Beta Coeff**  **(95% CI)** | **R-squared^b^** |
| --- | --- | --- | --- | --- |
| acetate | age, sex*, BMI | 621 | -1.46 (-4.29, 1.37) | 5.4% |
| acetoacetate | age*, sex*, BMI | 620 | -0.61 (-3.46, 2.24) | 2.4% |
| 3-hydroxybutyrate | age, sex, BMI* | 614 | -3.18 (-6.07, -0.28) | 2.7% |
| alanine | age, sex, BMI* | 621 | 0.10 (-3.0, 3.20) | 2.5% |
| glutamine | age*, sex*, BMI | 621 | -0.17 (-2.74, 2.40) | 28.6% |
| histidine | age, sex, BMI | 621 | 0.91 (-2.01, 3.82) | 0.8% |
| isoleucine | age, sex*, BMI* | 621 | 0.30 (-2.31, 2.90) | 28.8% |
| leucine | age, sex*, BMI* | 621 | -0.48 (-3.09, 2.12) | 16.9% |
| phenylalanine | age, sex*, BMI* | 621 | -1.34 (-3.81, 1.14) | 14.9% |
| tyrosine | age, sex*, BMI* | 617 | 0.96 (-1.57, 3.49) | 17.4% |
| valine | age, sex*, BMI* | 618 | -1.79 (-4.14, 0.56) | 39.0% |

Abbreviations: BMI=body mass index; CI=confidence interval

*this co-variate is also significant (p<0.05)

^a^biomarkers are all rank-transformed

^b^R-squared represents the variation explained from all variables in the model together

Supplemental Table 8. Linear regression models for 3-hydroxybutyrate as dependent variable and standardized MPNI p factor scores from different raters and ages as main independent variable

| **Biomarker^a^** | **Instrument-Rater-Age** | **Co-variates** | **N** | **Standardized**  **p Factor Beta Coeff**  **(95% CI)** |
| --- | --- | --- | --- | --- |
| 3-hydroxybutyrate | MPNI-Parent-12 | age, sex, BMI* | 676 | -3.29 (-21.81, 15.23) |
|  | MPNI-Teacher-12 | age, sex, BMI* | 677 | -20.45 (-39.33, -1.57) |
|  | MPNI-Teacher-14 | age, sex, BMI* | 542 | -12.58 (-34.45, 9.28) |
|  | MPNI-Self-14 | age, sex, BMI* | 672 | -20.74 (-38.20, -3.27) |
|  | MPNI-Co-twin-14 | age, sex, BMI* | 663 | -7.71 (-26.63, 11.20) |
|  | MPNI-Self-17 | age, sex, BMI* | 620 | -4.40 (-23.43, 14.63) |
|  | MPNI-Co-twin-17 | age, sex, BMI | 615 | 0.10 (-17.09, 17.29) |

Abbreviations: BMI=body mass index; CI=confidence interval; MPNI=Multidimensional Peer Nomination Inventory

*this co-variate is also significant (p<0.05)

^a^biomarkers are all rank-transformed
